# ETHNIC AND DEMOGRAPHIC DIFFERENCES IN THE UTILIZATION OF ANCILLARY HOSPICE SERVICES

**DOI:** 10.1093/geroni/igz038.483

**Published:** 2019-11-08

**Authors:** Anne D Halli-Tierney, Gregg Bell, Rebecca S Allen

**Affiliations:** 1 The University of Alabama College of Community Health Sciences, Tuscaloosa, Alabama, United States; 2 The University of Alabama, Tuscaloosa, Alabama, United States

## Abstract

Background: Research shows that ethnic differences exist in hospice service election, with fewer African American patients enrolling in hospice and having lower satisfaction with care compared to Non-Hispanic Whites. Hospice is interdisciplinary, with nursing care and “ancillary” services (social work, chaplain, nurses’ aide, volunteer). Little research exists about whether disparities exist in ancillary service election, and if patient demographics correlate with service utilization. We examined if ethnic or demographic differences exist among hospice beneficiaries in utilization of hospice services. Methods: Mixed-methods data collection took place from two community hospices. The quantitative arm involved retrospective chart review on new admissions from 2012 to 2016: acceptance of ancillary services and demographic data were collected as well as code status and outcome of hospice admission. The qualitative arm collected interview data from hospice personnel about thoughts on hospice care, which patients they think might decline ancillary services, and why. Results: Chart review was completed on 491 patient charts: interviews of hospice staff are ongoing. Sample was 55% female, 77% white, average age 77.8 years. Initial analysis on demographic data did not show statistical significance in utilization. Declination was lowest for social work (3.4% declined) and highest for hospice volunteer (88.9% declined). Initial interview themes involved need for patient education and role of health literacy. Discussion: Initial research does not show statistical ethnic or demographic differences in ancillary service utilization. However, broad utilization differences exist between services. Data can identify areas where hospices can improve care accessibility through patient education and personalization of services.

